# Supplementary material for: Large interannual variability in supraglacial lakes around East Antarctica
Source: Nat Commun. 2022 Mar 31;13:1711. doi: 10.1038/s41467-022-29385-3 (PMC8971459; doi:10.1038/s41467-022-29385-3)
Supplement: Supplementary file 1 — Supplementary Information [file 41467_2022_29385_MOESM1_ESM.pdf]

Large interannual variability in supraglacial lakes around East Antarctica (Supplementary Information)

Jennifer F. Arthur[1], Chris R. Stokes[1], Stewart S.R. Jamieson[1], J. Rachel Carr[2], Amber A. Leeson[3], Vincent Verjans[3]

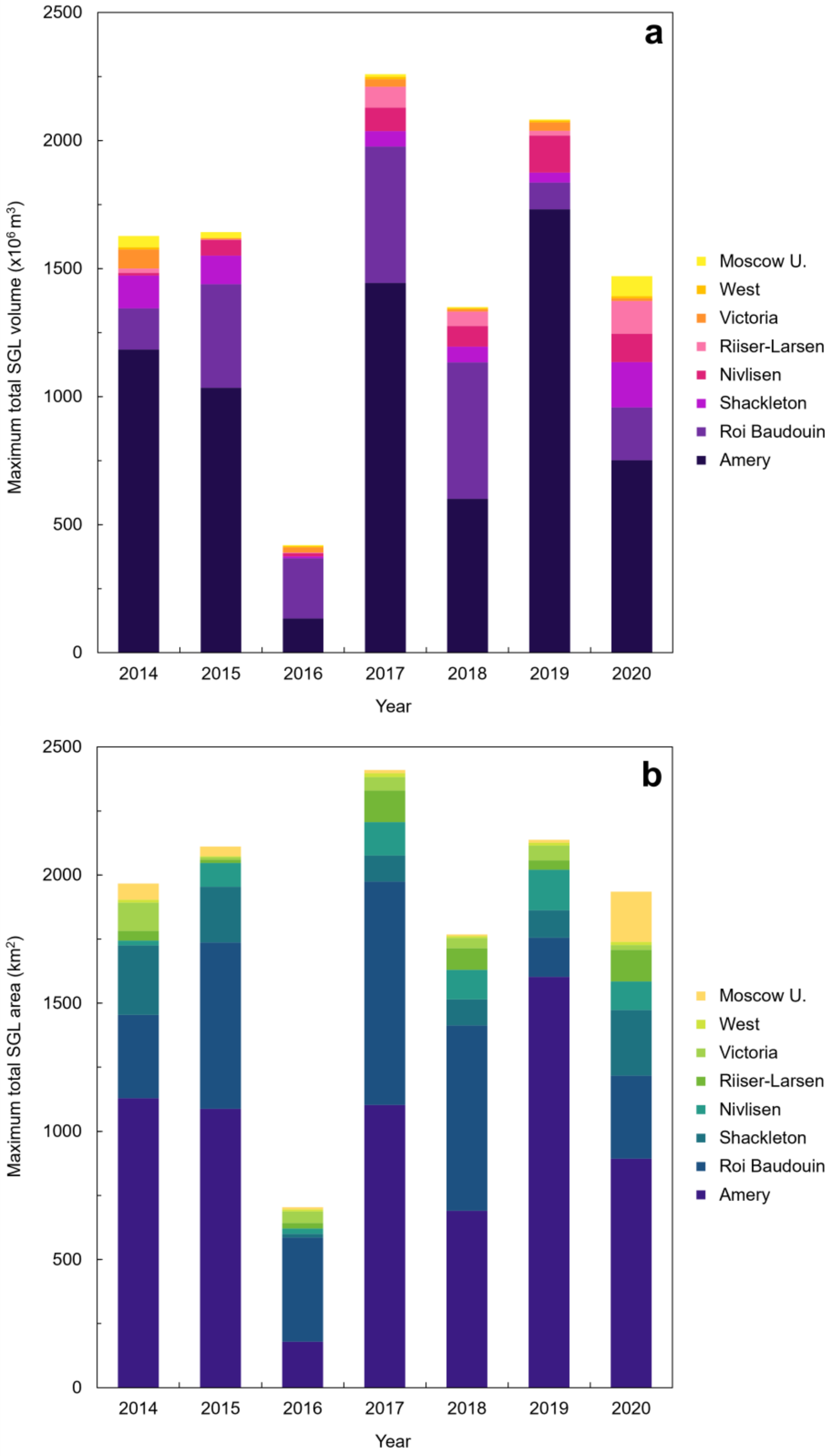

Supplementary Figure 1. Contributions of major ice shelves and regions to maximum total SGL volume (a) and area (b) around East Antarctica.

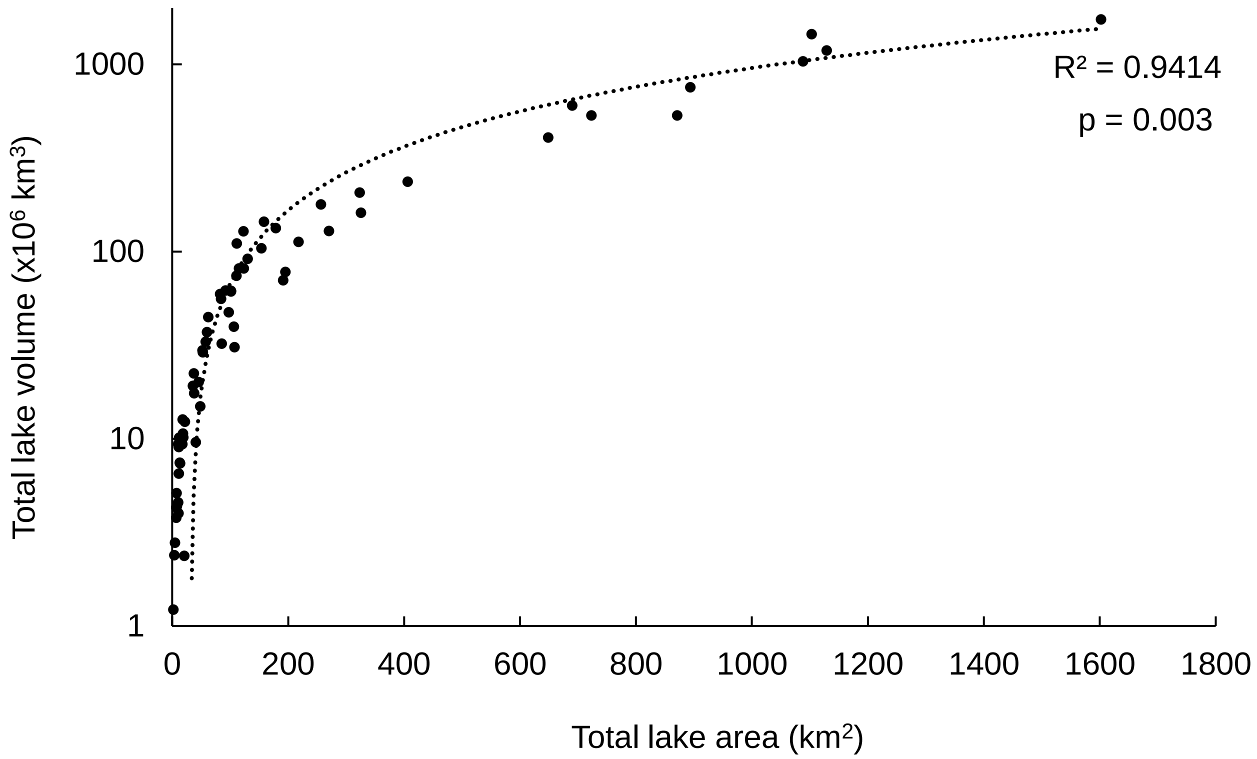

Supplementary Figure 2. Comparison between maximum January total supraglacial lake area and maximum January total lake volume on ice shelves (n = 8), outlet glaciers and/or coastal regions (n = 4) around East Antarctica from 2014 to 2020.

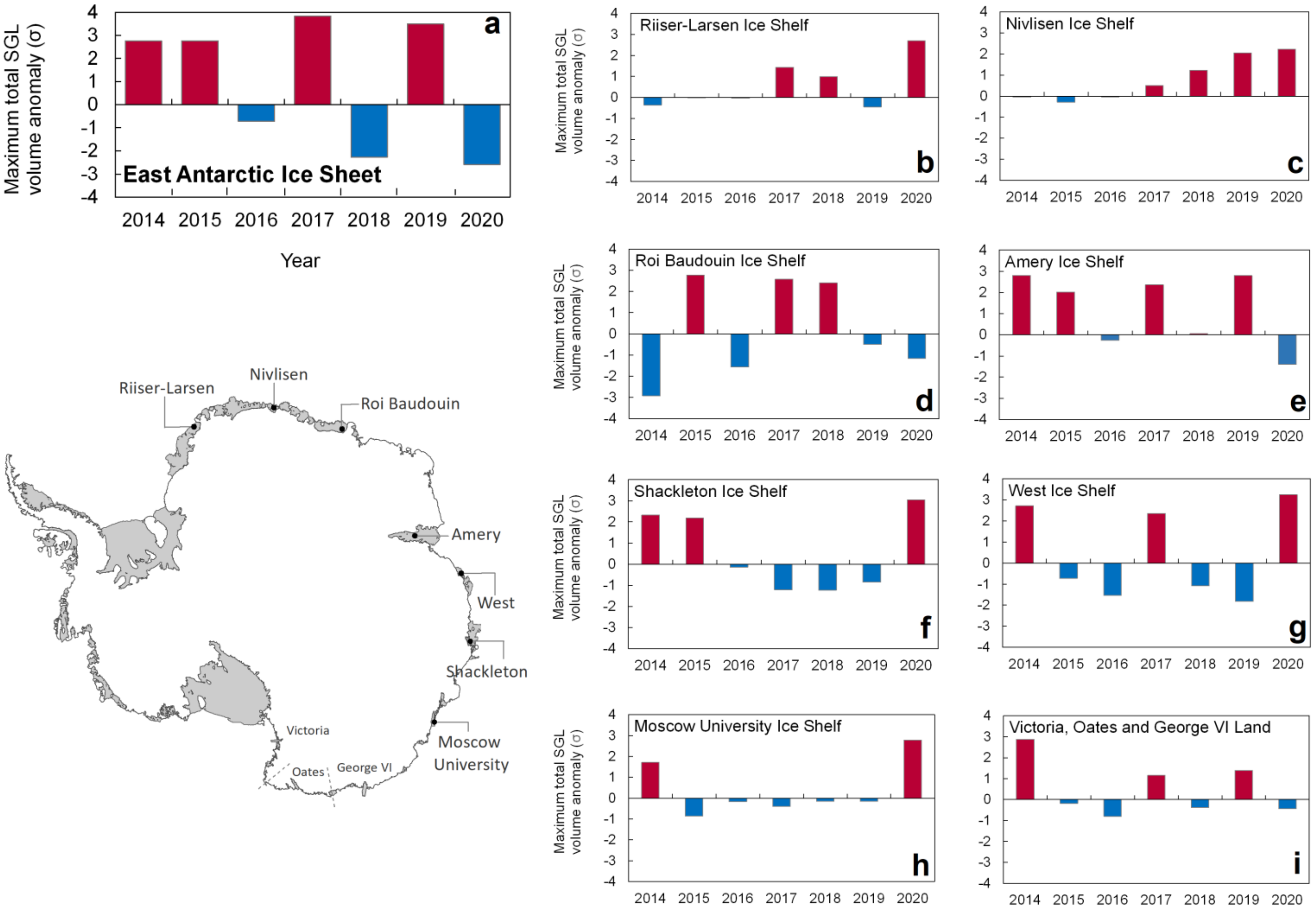

Supplementary Figure 3. (a) Relative total SGL volume anomalies (i.e. standard deviations from the average 2014-2020 maximum total lake volume) on the East Antarctic Ice Sheet. (b-i) Relative total SGL volume anomalies (i.e. standard deviations from the average 2014-2020 maximum total lake volume) on major ice shelves and regions. Grounding line from Rignot et al. (2016) and coastline from Mouginot et al. (2017).

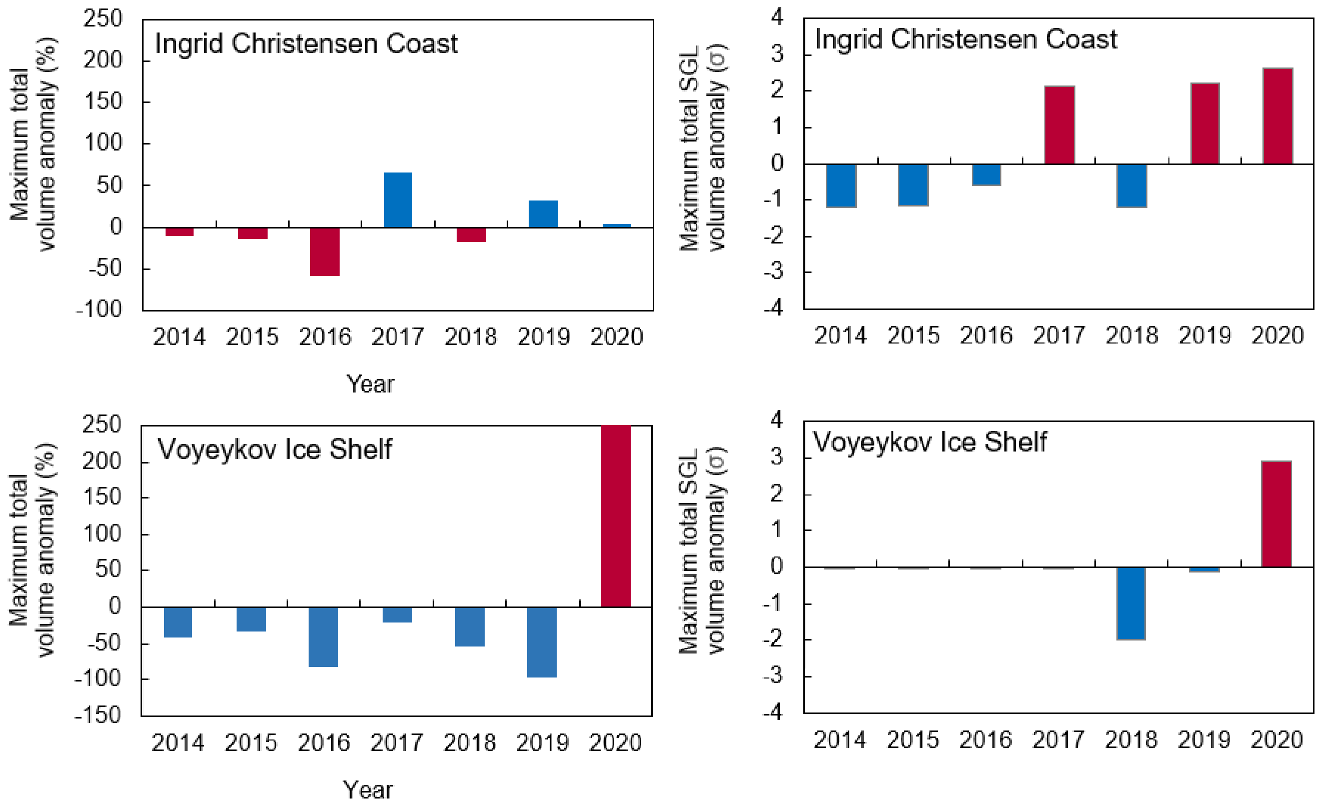

Supplementary Figure 4. Percentage SGL volume anomalies (i.e. percentages of the mean 2014-2020 maximum total lake volume) and anomalies as standard deviations for the Ingrid Christensen Coast region and Voyeykov Ice Shelf, East Antarctica.

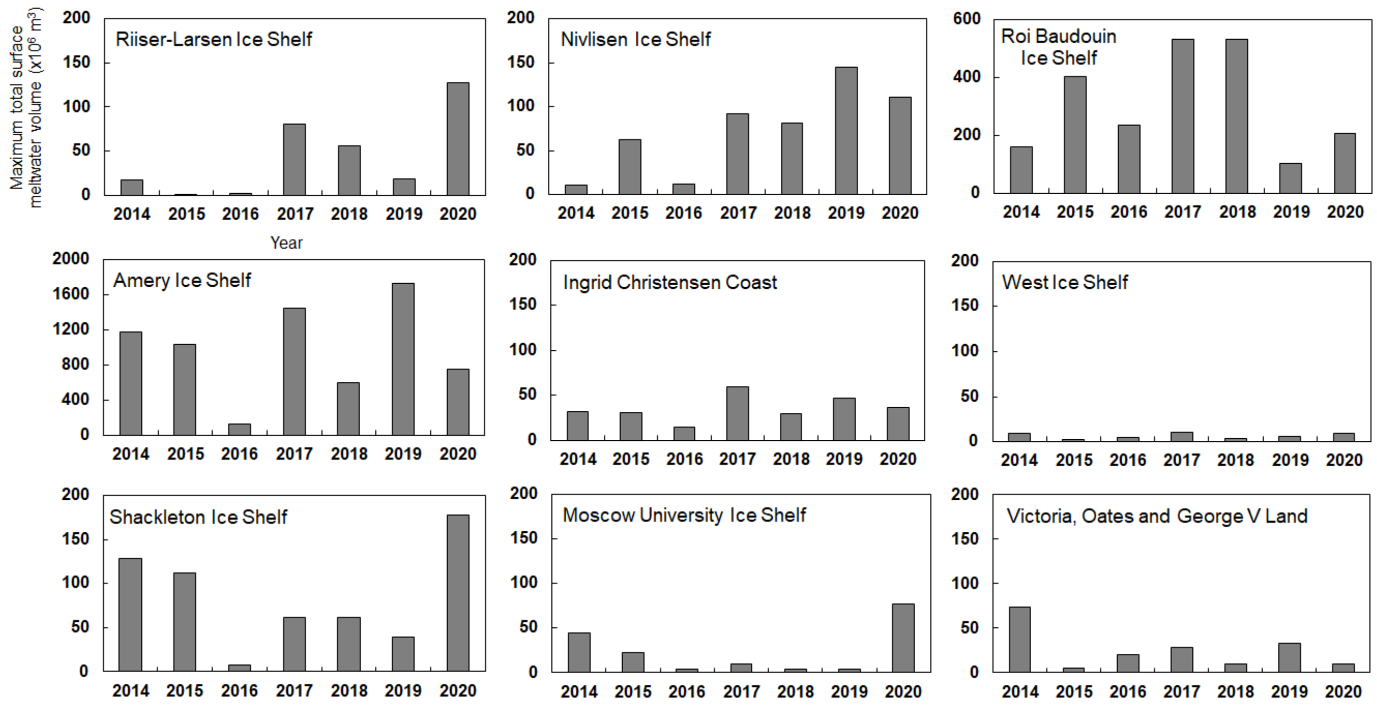

Supplementary Figure 5. Absolute total SGL volumes (in millions of cubic metres) on major ice shelves and regions of the East Antarctic Ice Sheet.

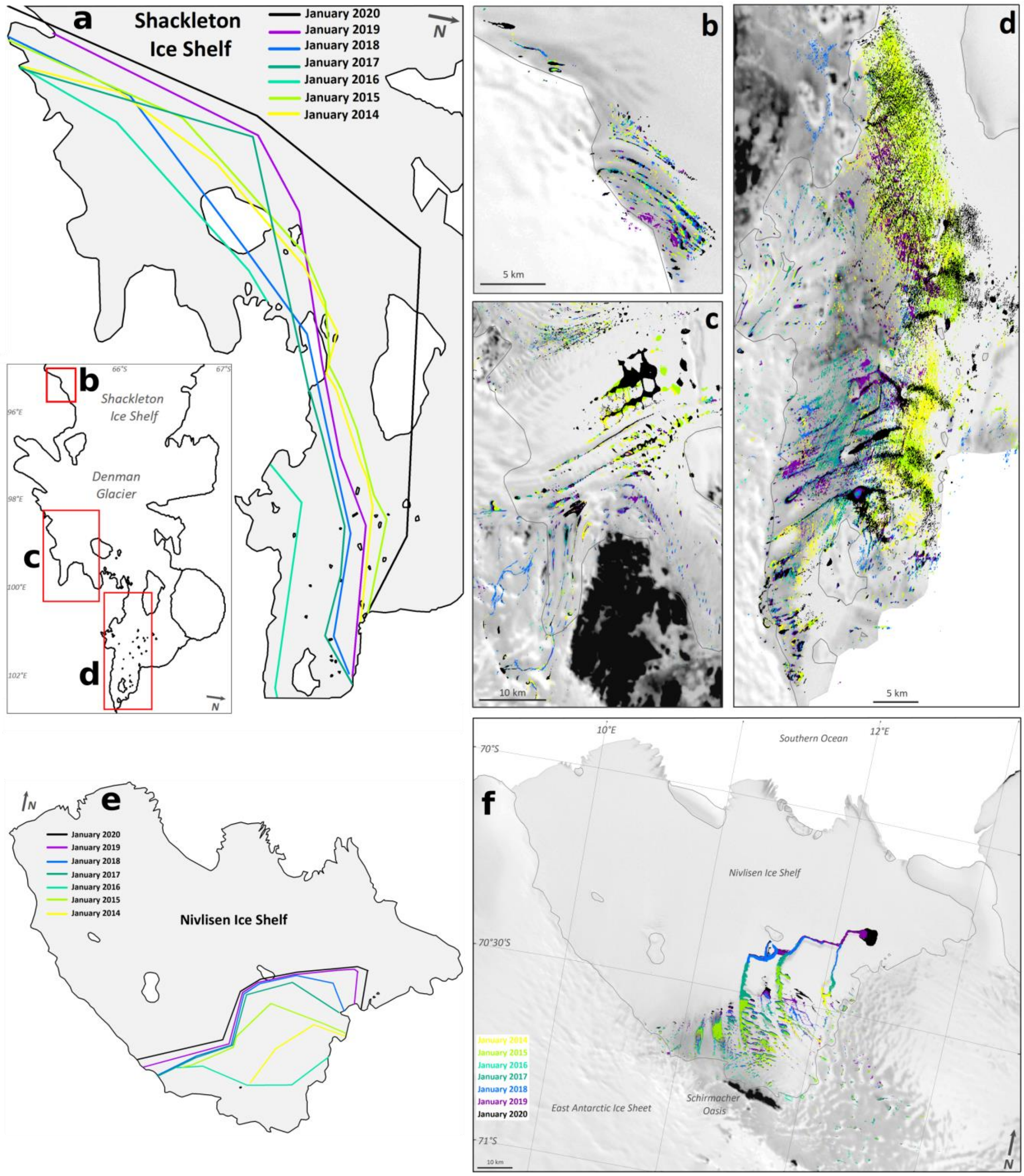

Supplementary Figure 6. Supraglacial lakes spreading towards the ice shelf calving fronts in more recent melt seasons on the Shackleton (a-d) and Nivlisen (e-f) ice shelves. Coloured bars in panels a and e denote the northern outer limit of lake-covered area. Black line denotes ice shelf outlines. Supraglacial lake extents are shown in the corresponding panels on the right. Grounding line from Rignot et al. (2016) and coastline from Mouginot et al. (2017).

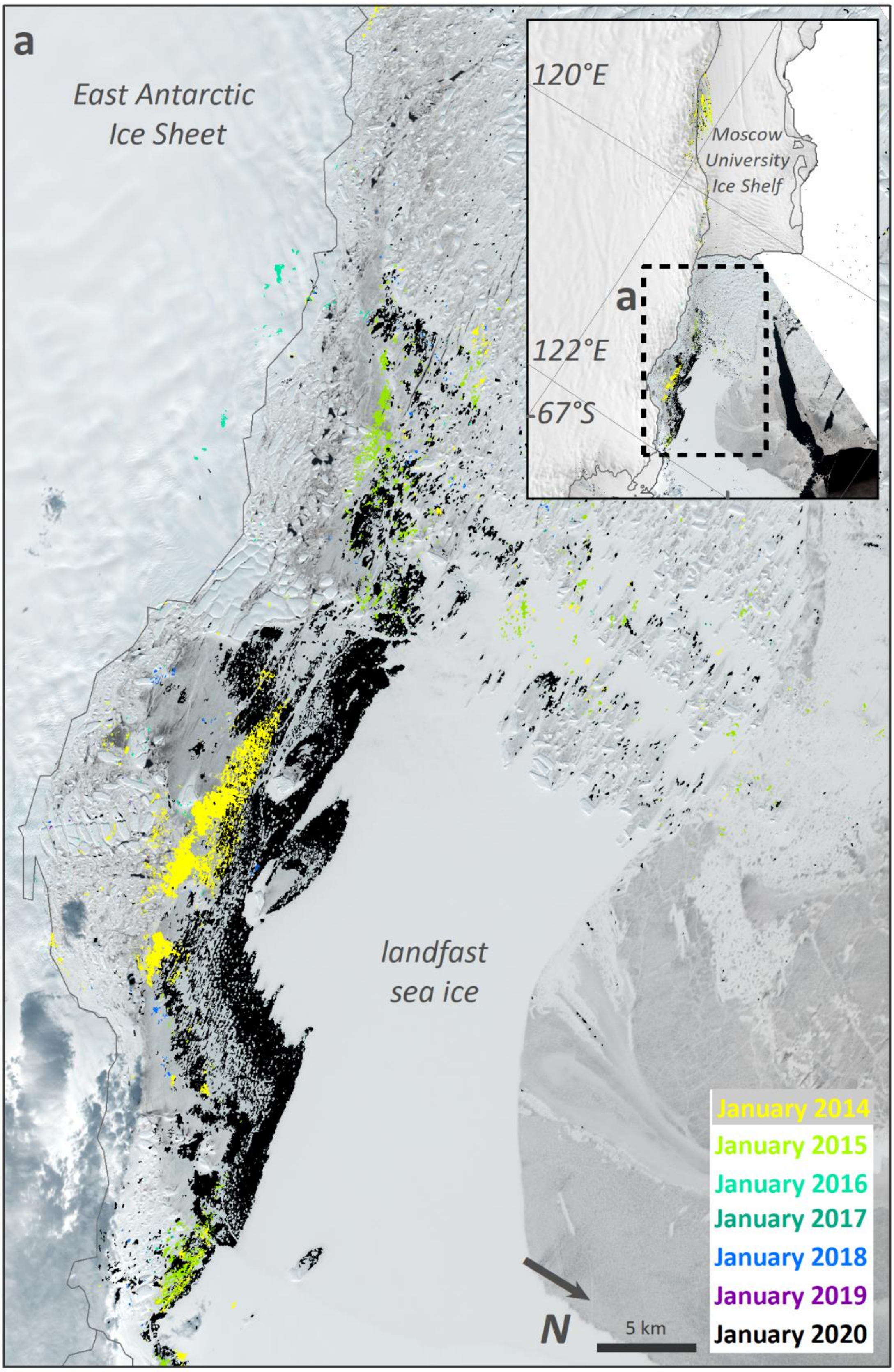

Supplementary Figure 7. Maximum January supraglacial lake extents showing SGL expansion on to landfast sea ice east of Moscow University Ice Shelf on 27th January 2020 (in black). Grounding line from Rignot et al. (2016) and coastline from Mouginot et al. (2017).

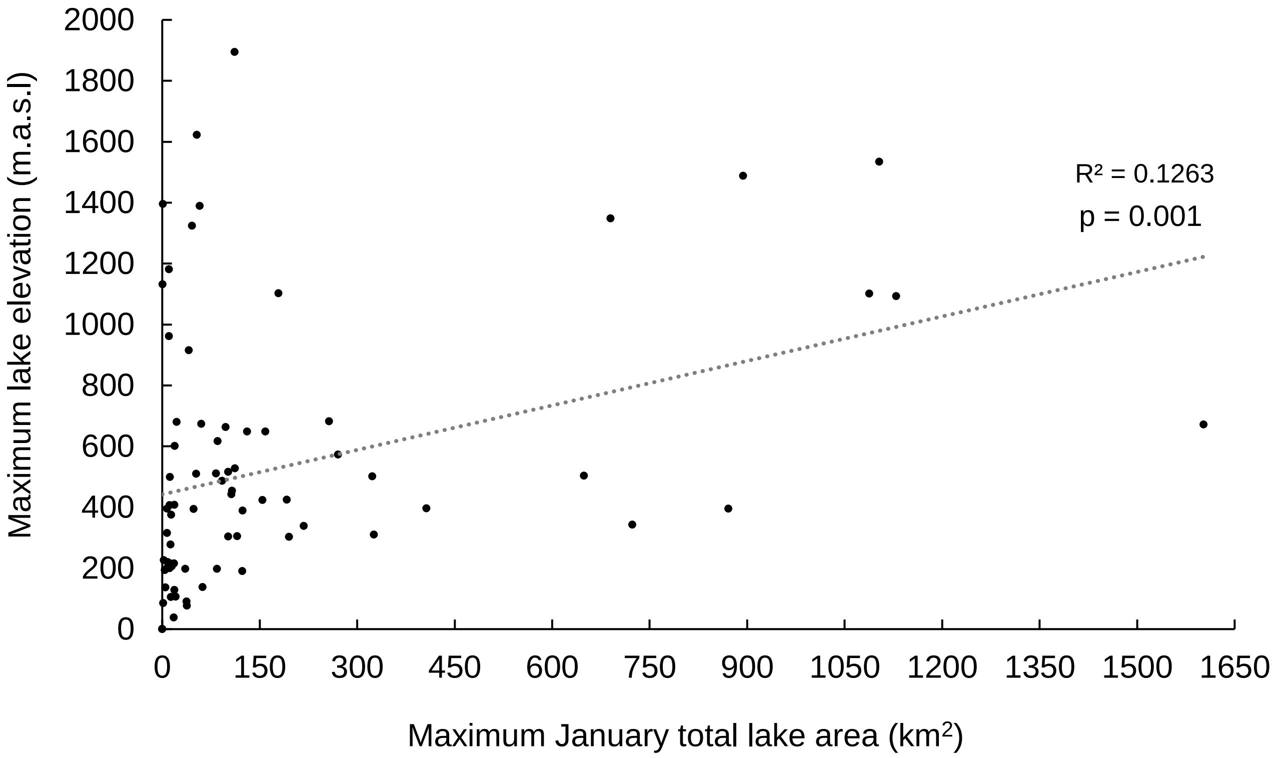

Supplementary Figure 8. Comparison between maximum January supraglacial lake area and maximum supraglacial lake elevation around East Antarctica.

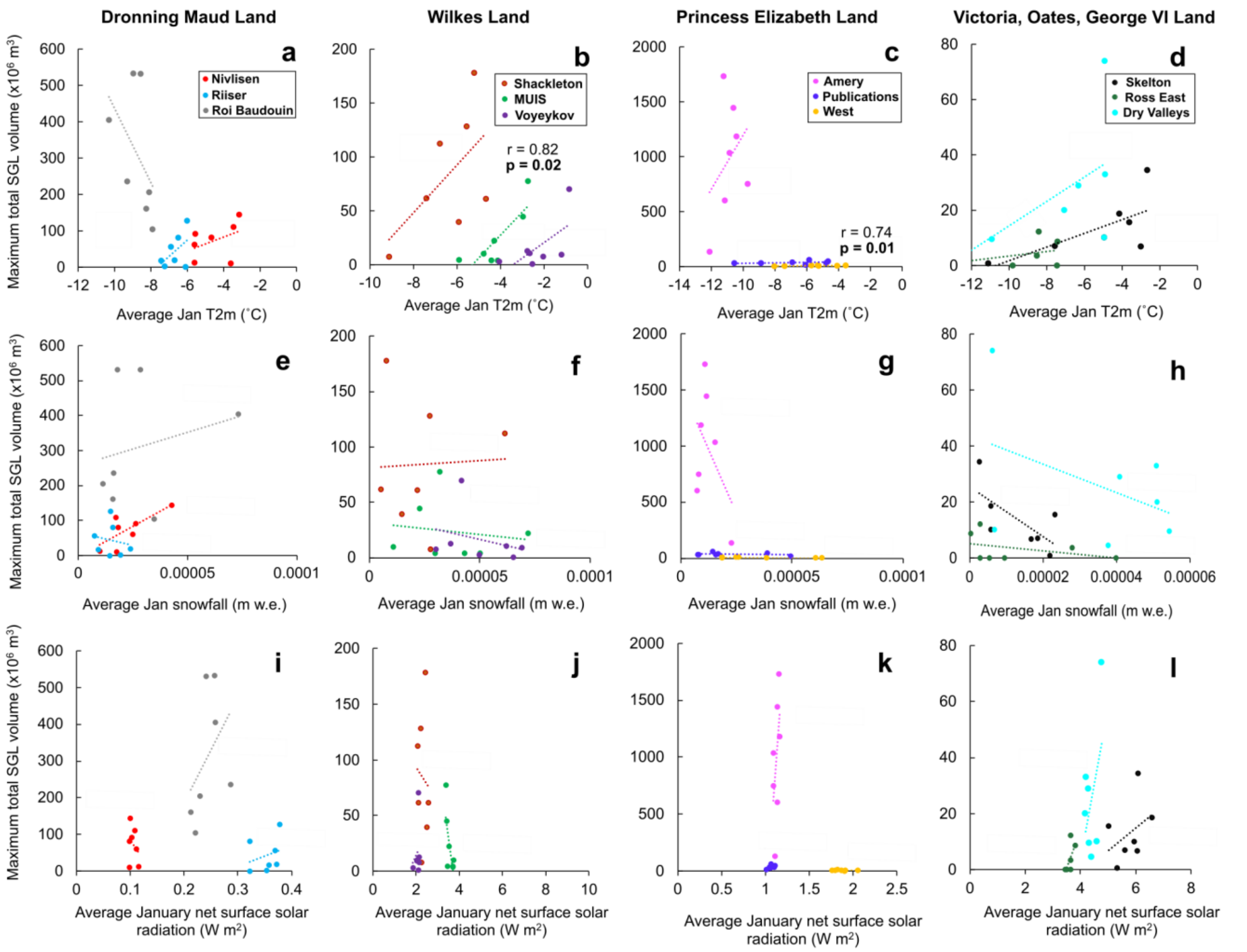

Supplementary Figure 9. Scatter plots of average January 2-m temperature (T_{2m}) (a-d), snowfall (e-h) and net surface solar radiation (S_{rad}) (i-l) from ice shelf grounding zones simulated by ERA5 reanalysis (see Methods) and maximum total SGL volume grouped by major EAIS region (columns). Individual ice shelves are represented by different colours (see Fig. 1 for locations). Significant relationships ($p < 0.05$) with a linear regression are displayed in bold.

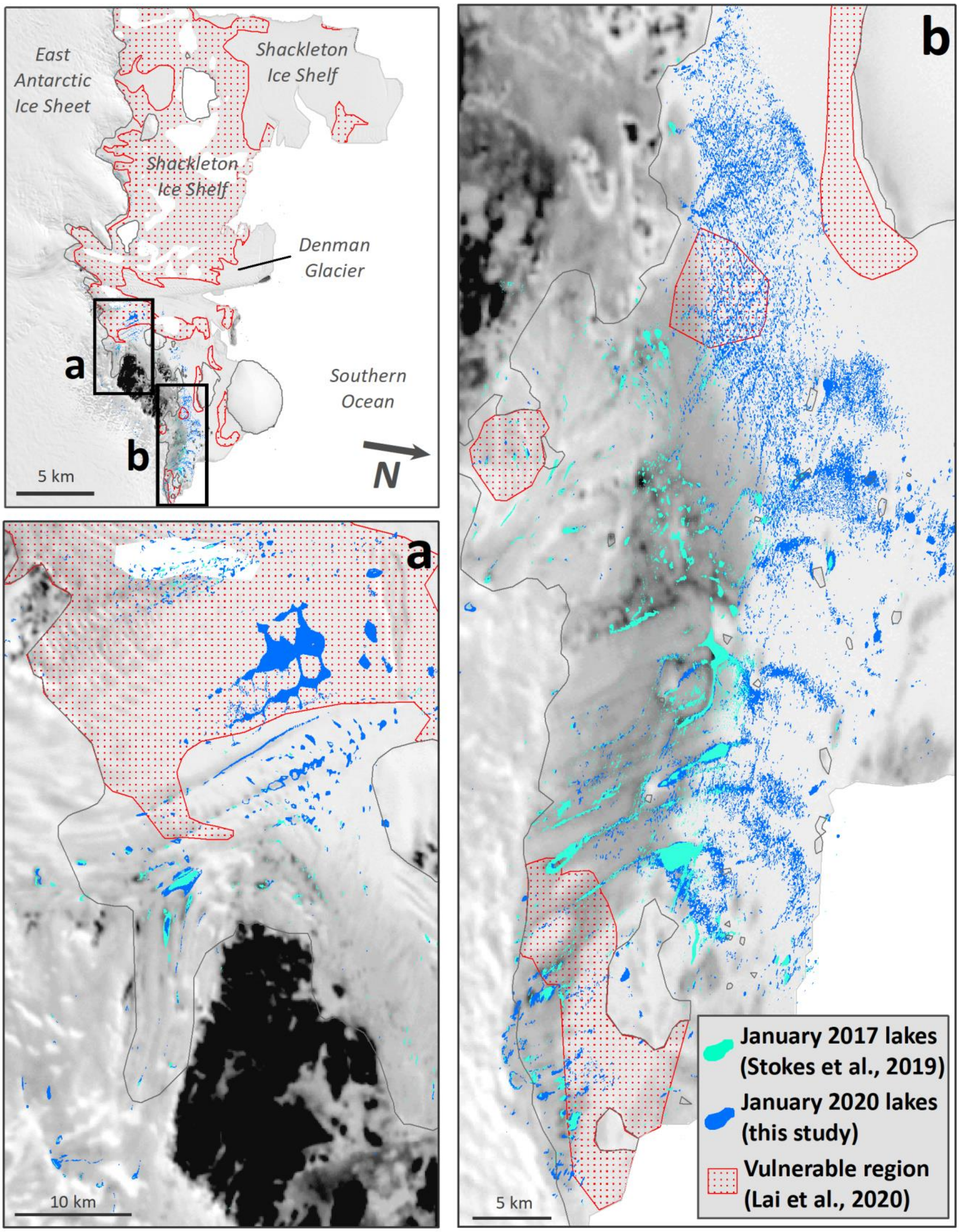

Supplementary Figure 10. Supraglacial lakes on Shackleton Ice Shelf in January 2017 (Stokes *et al.,* 2019) and in January 2020 (this study) overlaid on regions vulnerable to hydrofracture (i.e. where modelled surface fractures will be unstable if filled with meltwater when tensile resistive stresses exceed a critical threshold; Lai *et al.,* 2020). Grounding line from Rignot et al. (2016) and coastline from Mouginot et al. (2017).

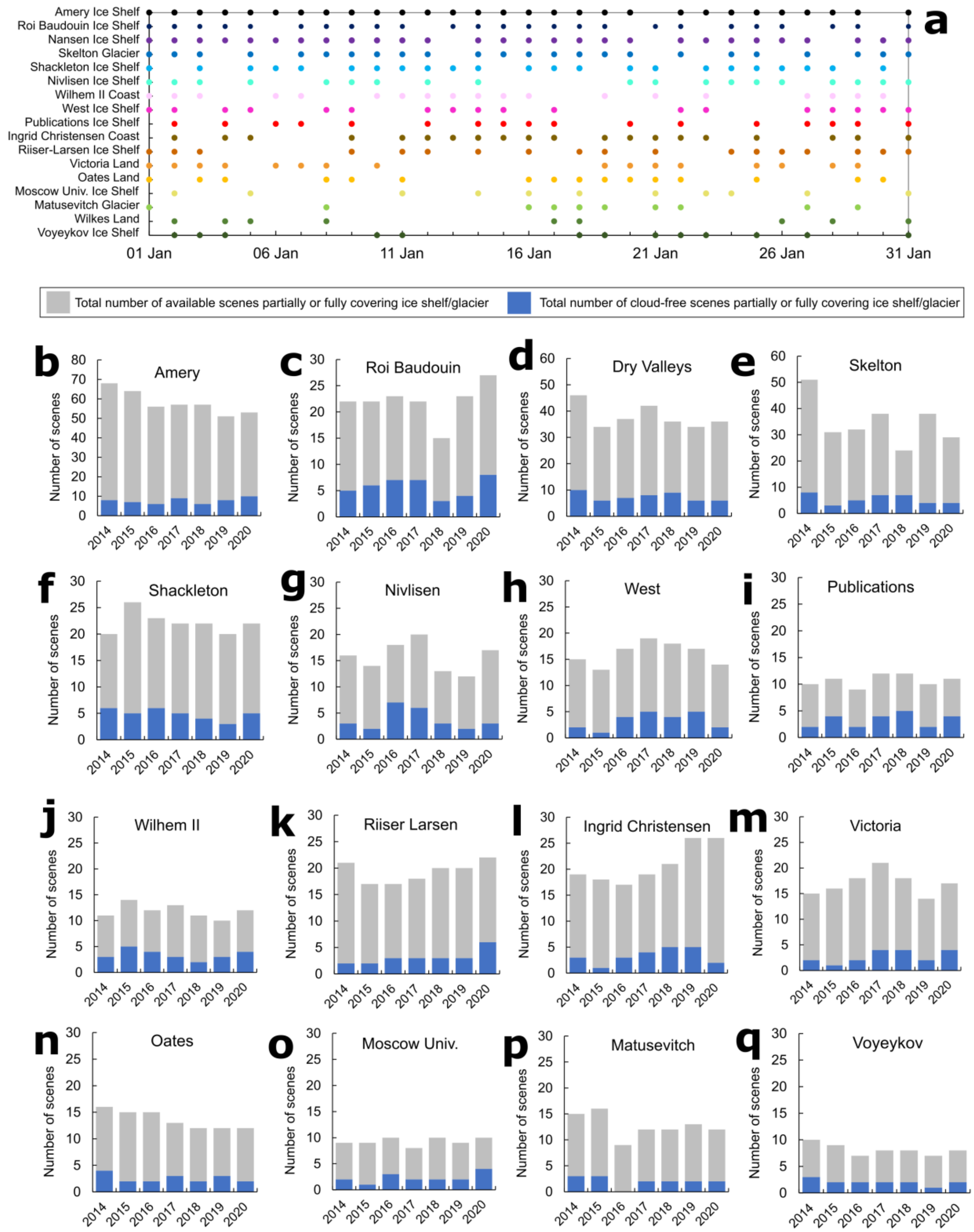

Supplementary Figure 11. (a) Imagery distribution on individual days within the month of January from 2014-2020 on major ice shelves and regions where lakes form around East Antarctica. One dot represents one day in January which has a useable Landsat 8 scene, where ‘useable’ is defined as cloud-free or mostly cloud-free scenes that may or may not contain lakes and does not include scenes dominated by cloud cover. (b-q) Frequency of Landsat 8 scenes for these locations in January from 2014 to 2020. Grey indicates total number of available scenes and blue indicates total number of useable (i.e. cloud-free) scenes.

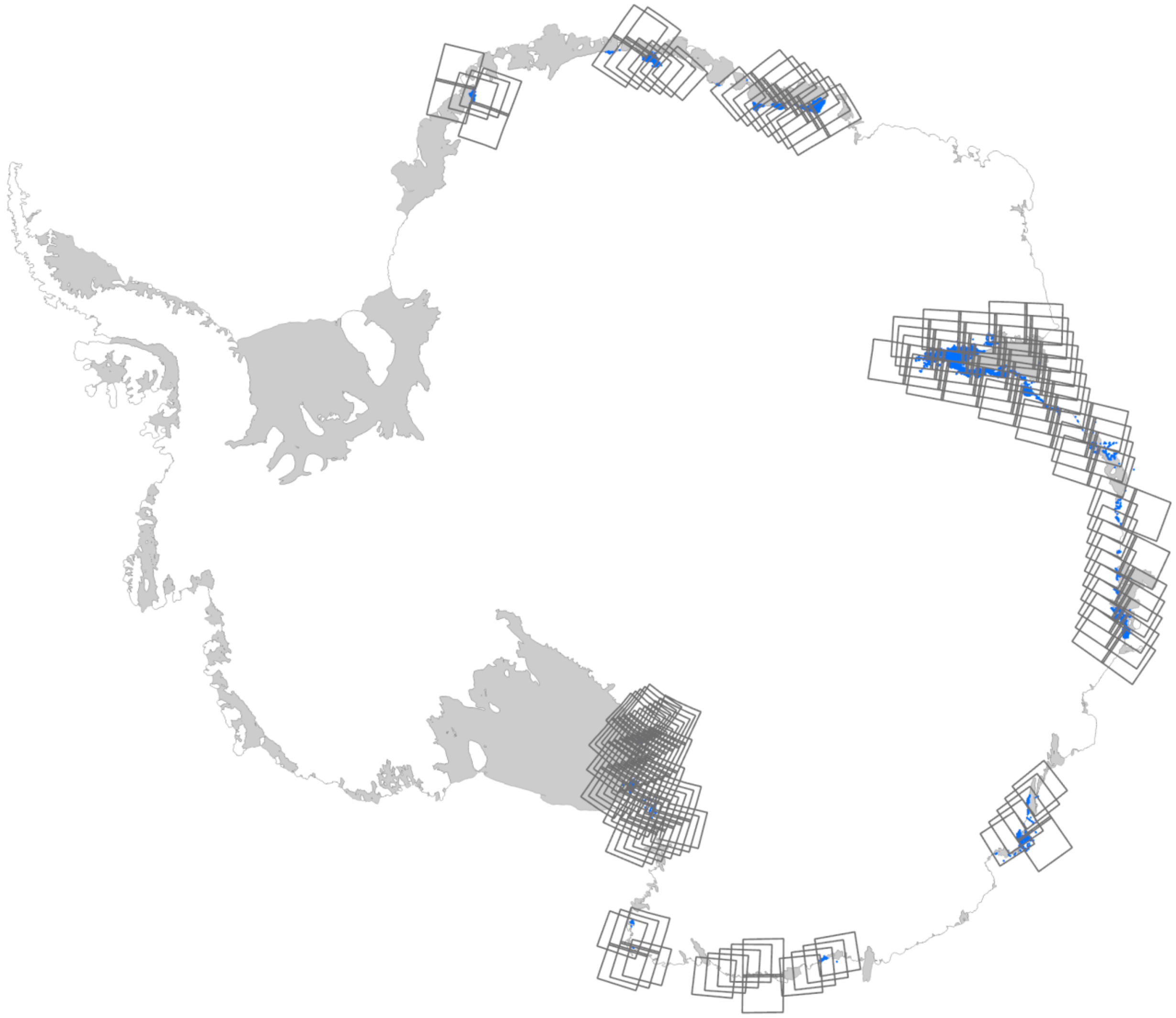

Supplementary Figure 12. Satellite imagery extents used in this study (i.e. all Landsat 8 tiles containing supraglacial lakes between January 2014 and 2020). Numbers of scenes for each ice sheet region are detailed in Supplementary Figure 9. Grounding line from Rignot et al. (2016) and coastline from Mouginot et al. (2017).

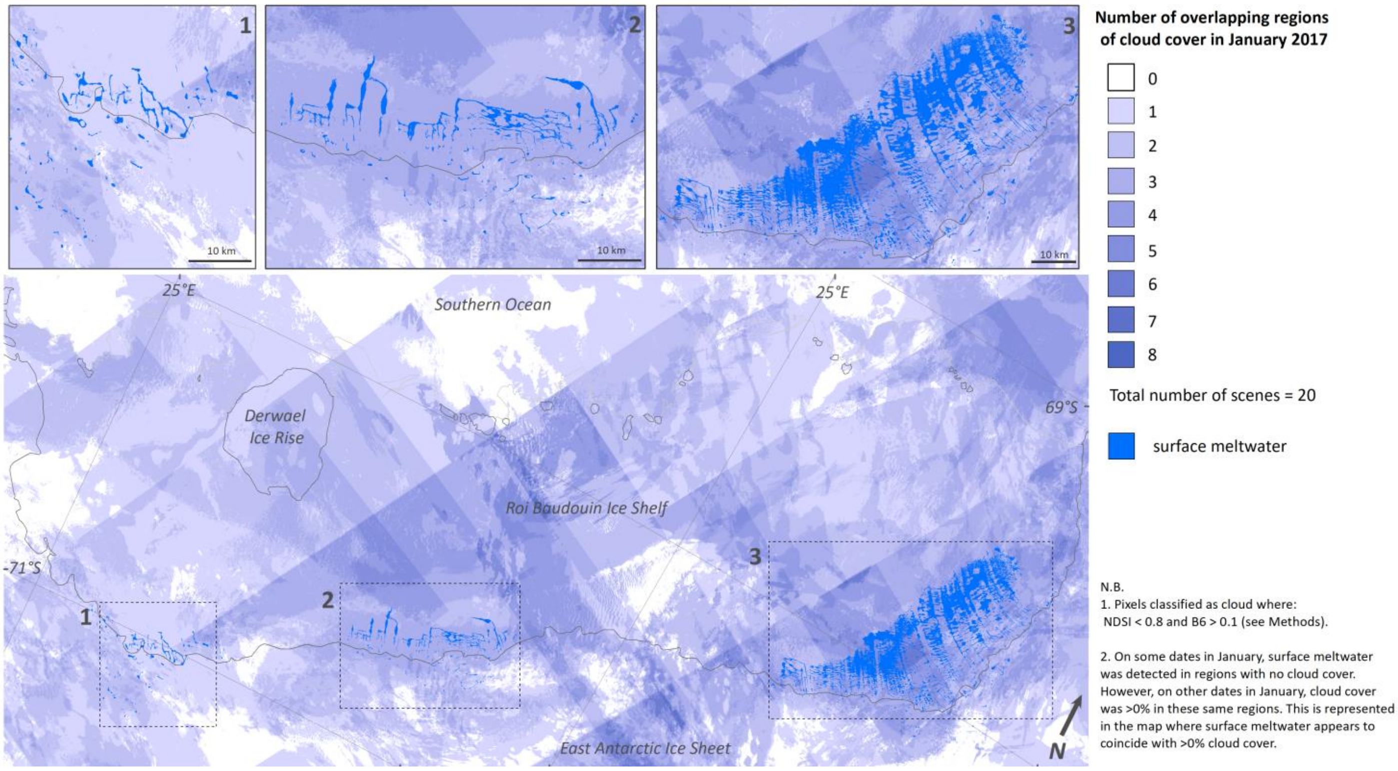

Supplementary Figure 13. Example of cloud cover mapping on Roi Baudouin Ice Shelf in January 2017. Extensive supraglacial lakes (in blue) are mapped despite cloud coverage in most satellite scenes. This demonstrates cloud cover does not prevent lakes being mapped even in regions that experience high cloud cover on some dates in January (darker purple shading). Grounding line from Rignot et al. (2016).

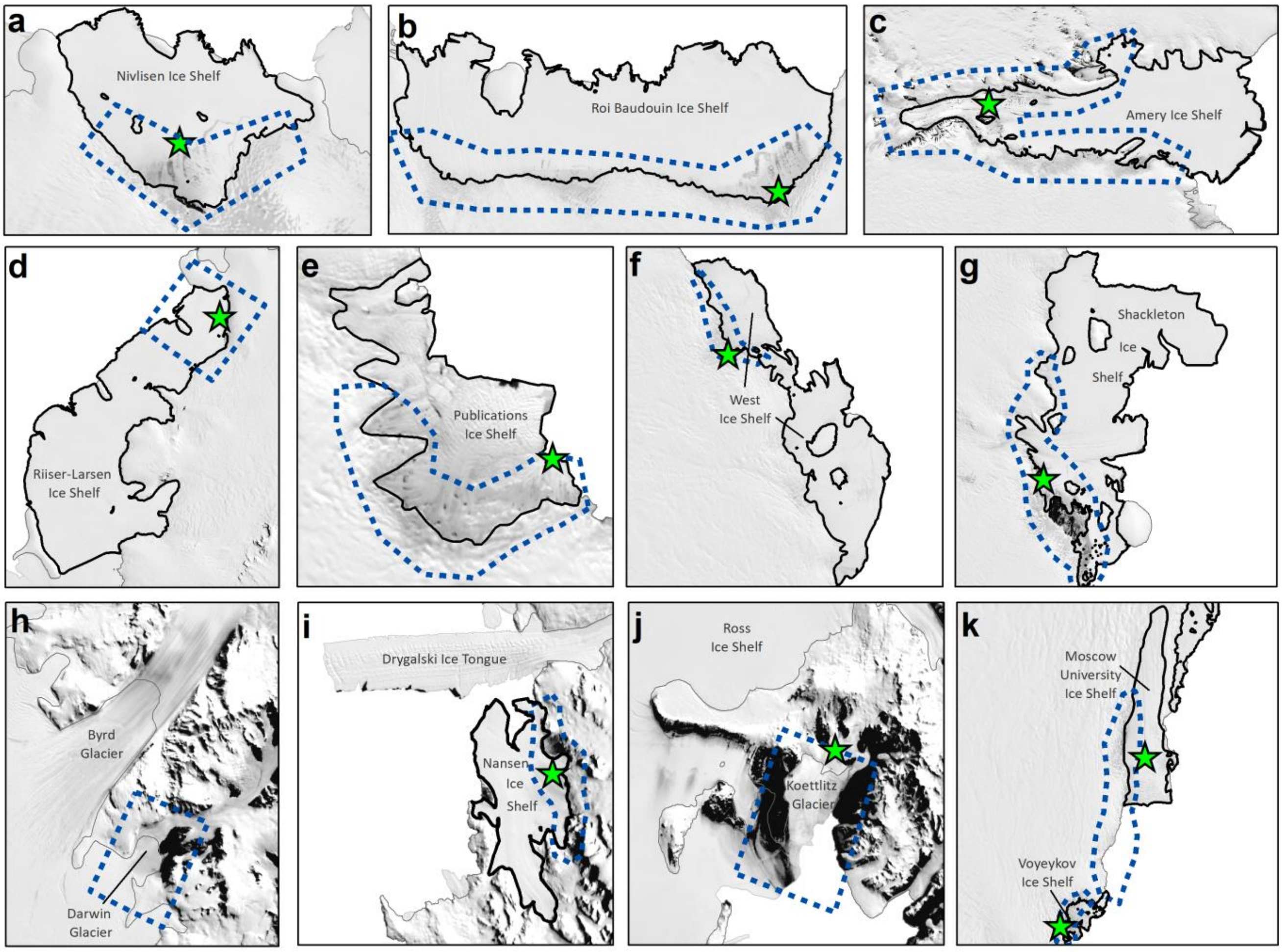

Supplementary Figure 14. ERA5 reanalysis extraction averaged within major East Antarctic ice shelf and outlet glacier grounding zones (dashed blue line), and locations of Community Firn Model extraction points (green stars) (a-k). ERA5 grid cells were only included in calculations of averages if their majority (i.e. midpoint) intersected with grounding zone polygons (see Supplementary Figure 12). Grounding line from Rignot et al. (2016) and coastline from Mouginot et al. (2017).

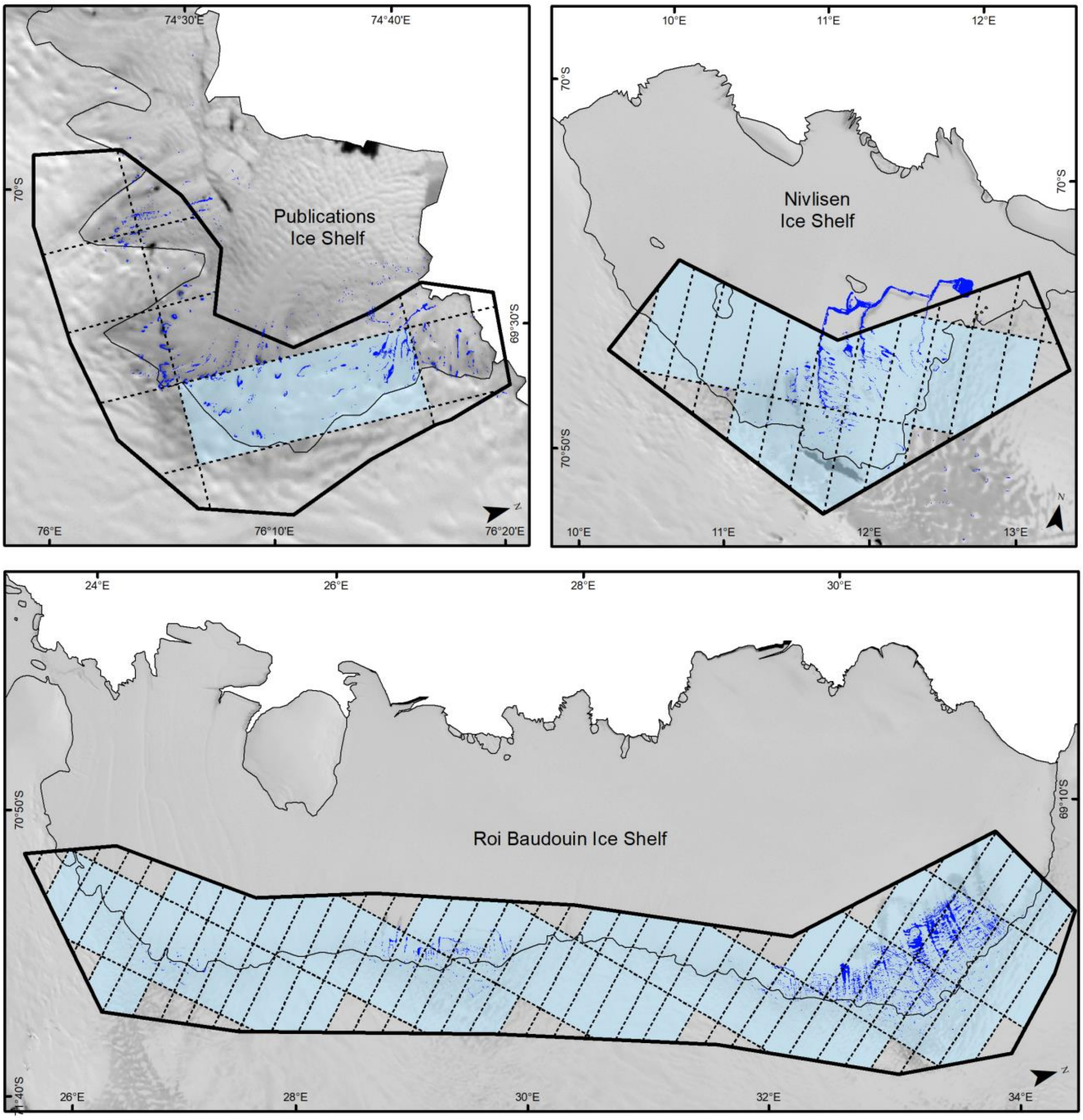

Supplementary Figure 15. Examples of ERA5 grid cells included in ice shelf grounding zone averages of 2-m temperature, surface temperature, snowfall and net solar radiation, shaded in pale blue. Black dashed lines indicate ERA5 grid cell boundaries and the black bold line indicates ice shelf grounding zone regions. Supraglacial lake outlines are shown in dark blue. Grounding line from Rignot et al. (2016) and coastline from Mouginot et al. (2017).

Supplementary Table 1. Results of regression analysis with variables simulated by ERA5 (left-hand column) using total lake area and total lake volume as explanatory variables. Significance at the 99% level is highlighted in green, and at the 95% level in blue. DJF = December-January-February. T_{2m} = 2-m air temperature, T_s = skin temperature, S_{rad} = net surface solar radiation. Winter snowfall refers to the February-December period preceding austral summer.

Univariate Linear Regression	R	R	R^2	R^2	p-value	
	Total Area	Total Volume	Total Area	Total Volume	Total Area	Total Volume
Mean January T_{2m}	-0.69	-0.65	0.48	0.42	1.9E-13	1.7E-11
Mean January T_{2m} (excluding the Roi Baudouin and Amery ice shelves)	-0.08	-0.07	0.007	0.006	0.47	0.52
Mean DJF T_{2m}	-0.72	-0.68	0.52	0.46	1.2E-13	8.6E-12
Mean DJF T_{2m} (excluding the Roi Baudouin and Amery ice shelves)	-0.18	-0.10	0.03	0.01	0.13	0.37
Mean January T_s	-0.64	-0.61	0.42	0.37	1.6E-10	3.8E-09
Mean DJF T_s	-0.66	-0.64	0.44	0.40	3.2E-11	5.7E-10
Mean January snowfall	-0.26	-0.26	0.06	0.06	0.02	0.02
Mean DJF snowfall	-0.34	-0.31	0.11	0.10	0.002	0.004
Mean preceding winter snowfall	-0.35	-0.34	0.12	0.11	0.001	0.002
Mean January S_{rad}	-0.36	-0.31	0.13	0.09	0.001	0.08
Mean DJF S_{rad}	-0.36	-0.31	0.14	0.09	0.001	0.005

Supplementary Table 2. Results of regression analysis with variables simulated by the CMF and MAR (left-hand column) using total lake area and total lake volume as explanatory variables. Significance at the 99% level is highlighted in green, and at the 95% level in blue. DJF = December-January-February. FAC = Firn air content.

Univariate Linear Regression	R	R	R^2	R^2	p-value	
	Total Area	Total Volume	Total Area	Total Volume	Total Area	Total Volume
Mean January FAC	-0.44	-0.37	0.19	0.13	5.1E-05	0.0008
Mean DJF FAC	-0.44	-0.37	0.19	0.13	5.4E-05	0.0009
Mean November FAC	-0.43	-0.36	0.18	0.12	6.50E-05	0.001
Mean January surface melt	0.26	0.19	0.06	0.03	0.02	0.09

Mean DJF surface melt	0.26	0.16	0.07	0.02	0.01	0.14
$\frac{\text{Mean November FAC}}{\text{Mean DJF surface melt}}$	-0.07	-0.05	0.004	0.003	0.54	0.60
Mean January minimum ice lens depth	-0.27	-0.24	0.07	0.05	0.01	0.03
Mean DJF minimum ice lens depth	-0.26	-0.24	0.07	0.05	0.01	0.03
Mean January runoff	0.19	0.07	0.03	0.005	0.08	0.53
Mean DJF runoff	0.21	0.08	0.04	0.006	0.06	0.48

Supplementary Table 3. Results of regression analysis for individual ice shelves with variables simulated by the CMF and MAR (mean November firn air content-to-DJF melt, mean January surface runoff, mean January total surface melt and depth of shallowest ice lens) using total lake volume as the explanatory variable. Significance relationships ($p < 0.05$) are indicated in blue.

**Ice shelf**	**Nov FAC: DJF melt** (R)	**Nov FAC: DJF melt** (R^2)	**Jan melt** (R)	**Jan melt** (R^2)	**DJF melt** (R)	**DJF melt** (R^2)	**Jan runoff** (R)	**Jan runoff** (R^2)	**DJF runoff** (R)	**DJF runoff** (R^2)	**Jan Ice Lens** (R)	**Jan Ice Lens** (R^2)
Nivlisen	-0.55	0.28	0.46	0.21	0.54	0	0	0	0	0	0.20	0.04
Riiser Larsen	-0.51	0.43	0.75	**0.57**	0.67	0.46	0.76	**0.58**	0.76	0.58	-0.57	0.12
Roi Baudouin	0.28	0.31	-0.04	0.002	-0.17	0.02	-0.04	0.002	-0.22	0.05	-0.35	0.32
Shackleton	-0.65	**0.85**	0.73	0.54	0.84	0.70	0.86	**0.75**	0.91	0.84	-0.67	0.46
Moscow Univ.	-0.60	**0.79**	0.94	**0.88**	0.87	0.76	0	0	0	0	-0.31	0.09
Voyeykov	-0.59	**0.87**	0.84	**0.70**	0.86	0.74	0	0	0	0	-0.45	0.21
Amery	-0.42	0.52	0.66	0.44	0.70	0.50	0	0	0	0	0.05	0.003
Publications	-0.59	0.50	0.44	0.49	0.47	0.22	0.07	0.006	0.06	0.004	-0.09	0.008
West	-0.39	0.75	0.70	0.19	0.64	0.41	0	0	0	0	-0.01	0.006
Skelton	-0.41	0.21	-0.02	0.0002	0.30	0.10	0	0	0	0	-0.40	0.08
Ross East	-0.49	0.46	0.76	0.007	0.54	0.29	0	0	0	0	-0.08	0.007
Dry Valleys	-0.45	0.38	0.56	0.31	0.60	0.36	0	0	0	0	-0.51	0.26

Supplementary Table 4. Results of regression analysis for individual ice shelves with variables simulated by ERA5 reanalysis (mean December-January-February (DJF) 2-m temperature (T_{2m}), mean January 2-m temperature, mean snowfall in the preceding winter (February to December), mean January snowfall, mean DJF net surface solar radiation (S_{rad}) and mean January net surface solar radiation using total lake volume as the explanatory variable. Significance relationships ($p < 0.05$) are indicated in bold.

Ice shelf	DJF T_{2m} (R)	DJF T_{2m} (R^2)	Jan T_{2m} (R)	Jan T_{2m} (R^2)	Preceding winter snowfall (R)	Preceding winter snowfall (R^2)	Jan snowfall (R)	Jan snowfall (R^2)	DJF S_{rad} (R)	DJF S_{rad} (R^2)	Jan S_{rad} (R)	Jan S_{rad} (R^2)
Nivlisen	0.52	0.62	0.42	0.17	-0.57	0.22	0.74	0.61	0.24	0.04	-0.3	0.56
Riiser Larsen	0.67	**0.70**	0.51	0.30	-0.36	0.10	-0.17	0.11	-0.64	0.38	0.27	0.09
Roi Baudouin	-0.41	0.16	-0.48	0.23	0.34	0.24	0.22	0.02	0.62	0.28	0.41	0.07
Shackleton	0.26	0.14	0.57	0.33	-0.47	0.26	0.04	0.01	0.02	0.01	-0.09	0.01
Moscow Univ.	0.44	0.13	0.82	**0.67**	-0.63	0.44	-0.14	0.07	-0.51	0.33	-0.74	0.50
Voyeykov	0.37	0.18	0.64	0.41	-0.61	0.39	-0.30	0.01	0.12	0.01	0.17	0.02
Amery	0.65	0.61	0.34	0.11	-0.51	0.44	-0.46	0.19	0.84	**0.80**	0.56	0.33
Publications	0.73	0.61	-0.18	0.03	-0.81	0.66	-0.28	0.12	0.53	0.43	0.52	0.37
West	0.79	**0.79**	0.86	**0.74**	-0.02	0.02	-0.36	0.06	0.06	0.01	0.18	0.06
Skelton	0.20	0.07	0.31	0.09	-0.28	0.20	-0.67	0.20	0.60	0.12	0.33	0.14
Ross East	0.65	0.1	0.76	0.58	-0.31	0.01	-0.67	0.06	0.10	0.02	0.31	0.34
Dry Valleys	0.23	0.20	0.58	0.33	0.03	0.03	-0.43	0.13	0.52	0.25	0.48	0.42

Supplementary Table 5. Details of Landsat 8 imagery in Figure 6.

Location	Date	Scene Identifier
Nivlisen Ice Shelf	14/1/2020	LC08_L1GT_165110_20200114_20200127_01_T2
Roi Baudouin Ice Shelf	11/1/2018	LC08_L1GT_154109_20180111_20180119_01_T2
Riiser Larsen Ice Shelf	16/1/2020	LC08_L1GT_179111_20200116_20200127_01_T2
Moscow University Ice Shelf	16/1/2020	LC08_L1GT_099107_20200116_20200127_01_T2
Shackleton Ice Shelf	3/1/2014	LC08_L1GT_111107_20140103_20170427_01_T2
Shackleton Ice Shelf	27/1/2017	LC08_L1GT_111106_20170127_20170214_01_T2
Amery Ice Shelf	17/1/2019	LC08_L1GT_127111_20190117_20190131_01_T2
Publications Ice Shelf	14/1/2014	LC08_L1GT_124109_20140114_20170426_01_T2
Nansen Ice Shelf	3/1/2014	LC08_L1GT_061113_20140104_20170427_01_T2
Koettlitz Glacier	3/1/2014	LC08_L1GT_054116_20140103_20170427_01_T2
